# The mutational landscape of the adult healthy parous and nulliparous human breast

**DOI:** 10.1038/s41467-023-40608-z

**Published:** 2023-09-06

**Authors:** Biancastella Cereser, Angela Yiu, Neha Tabassum, Lisa Del Bel Belluz, Sladjana Zagorac, Kenneth Russell Zapanta Ancheta, Rongrong Zhong, Cristian Miere, Alicia Rose Jeffries-Jones, Nina Moderau, Benjamin Werner, Justin Stebbing

**Affiliations:** 1 Cancer Genetics Group, Department of Surgery and Cancer, Imperial College London, London, UK; 2 Evolutionary Dynamics Group, Centre for Cancer Genomics and Computational Biology, Barts Cancer Institute, Queen Mary University of London, London, UK; 3 Department of Life Sciences, Anglia Ruskin University (ARU), Cambridge, UK; 4 Present Address: Molecular Oncology Programme, Growth Factors, Nutrients and Cancer Group, Centro Nacional de Investigaciones Oncológicas, Madrid, Spain; 5 Present Address: Pathobiology and Population Sciences, Royal Veterinary College, Hatfield, UK

**Keywords:** Cancer genetics, Breast cancer, Genome

## Abstract

The accumulation of somatic mutations in healthy human tissues has been extensively characterized, but the mutational landscape of the healthy breast is still poorly understood. Our analysis of whole-genome sequencing shows that in line with other healthy organs, the healthy breast during the reproduction years accumulates mutations with age, with the rate of accumulation in the epithelium of 15.24 ± 5 mutations/year. Both epithelial and stromal compartments contain mutations in breast-specific driver genes, indicative of subsequent positive selection. Parity- and age-associated differences are evident in the mammary epithelium, partly explaining the observed difference in breast cancer risk amongst women of different childbearing age. Parity is associated with an age-dependent increase in the clone size of mutated epithelial cells, suggesting that older first-time mothers have a higher probability of accumulating oncogenic events in the epithelium compared to younger mothers or nulliparous women. In conclusion, we describe the reference genome of the healthy female human breast during reproductive years and provide evidence of how parity affects the genomic landscape of the mammary gland.

## Abstract (web summary)

While many tissues have been investigated for natural somatic mutations, human breast tissue has not been well studied. Here, the authors characterize somatic mutations in human breast tissue, finding effects of age and parity.

## Introduction

In all adult healthy tissues, somatic mutations increase through adult life^1–10^, but the mutational landscape of the human breast has not been characterized thus far.

Compared to other solid tissues, the adult human breast is characterized by a generally slow proliferating epithelium, which undergoes significant changes during reproduction, and in particular during pregnancy. It has been estimated that the gain of 0.7 ± 0.1 new cells per day in pre-menopausal women^11^, but the proliferation index can increase nearly 5 times during pregnancy^12^. Furthermore, after breast-feeding, a strong apoptotic drive, which determines the beginning of post-partum mammary involution^13^, contributes to major tissue remodelling which can last up to 10 years after parturition^14^.

Several studies have recognized the complex contribution of parity and age to the healthy mammary genome, including to the risk of breast cancer (BC) development^15–17^. Compartment-specific sequencing of healthy tissue is therefore crucial not only to understand the architecture of the normal breast but also to determine the probability of developing tissue-associated diseases, including BC. However, pregnancy-specific mutational changes or clonal dynamics of the healthy breast, and in particular of cancer-associated genes are still unknown.

In this study, we sequence the whole genome of the healthy breast samples during reproductive years and determine how both age and pregnancy alter the genomic composition of the mammary gland, both in the epithelial and stromal compartments, and the implications on cancer development.

## Results

### Sample source and sequencing

The most common source of research material for studies on the non-diseased breast is reduction mammoplasty, which is often characterized by a higher body mass index (BMI) of the donor. While a higher BMI has been associated with a higher risk of developing BC in postmenopausal women^18^, it confers a protection towards the malignancy in younger individuals^19^. The contribution of body-mass index (BMI) to the alteration of the genetic landscape of mammoplasty specimens is complex and not usually acknowledged and highlights the need for different sources of healthy breast tissue for research.

To avoid a bias for different BMIs in the sample cohort, we chose the open-access Susan G. Komen Tissue Bank at IU Simon Cancer Center, which provided us with single ~0.5 cm^2^ biopsies collected by healthy donors. These specimens are characterized by less material than a reduction mammoplasty, thus not allowing multi-sampling. However, the samples are provided with a full record of parity and clinical history, including cancer history and BMI status, making them the preferred source of tissue for this study.

**Fig. 1:**
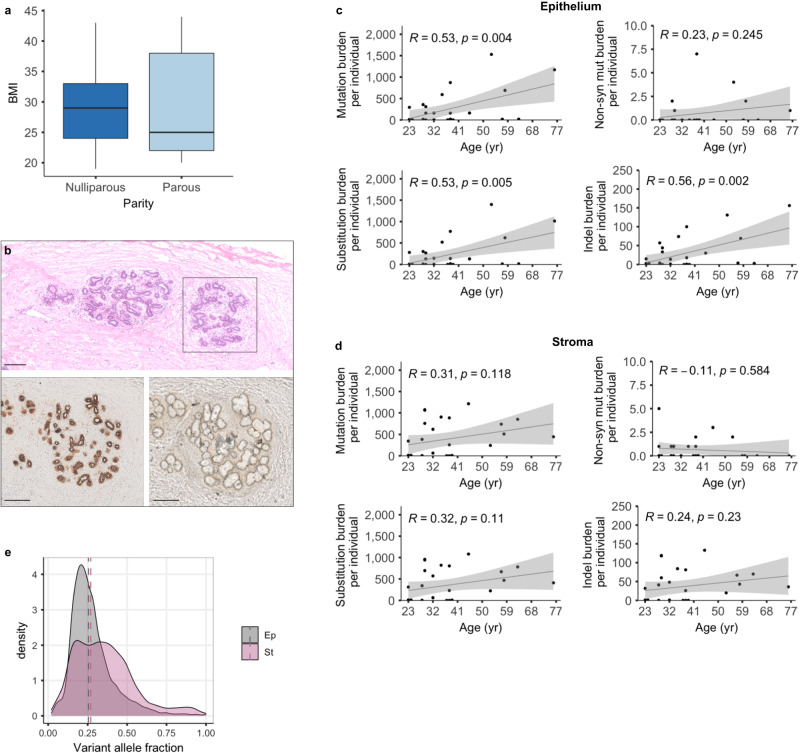
Mutational burden of the mammary gland. **a** Average BMI of sample groups (nulliparous = 12, parous = 17). The box extends from the 25th to the 75th percentile (Q1 to Q3) with a line at the median. Lines extending from both ends of the box indicate variability outside Q1 and Q3. The minimum/maximum whisker values are calculated as Q1/Q3 -/ + 1.5 * IQR (Interquartile range, defined as the range of values between Q1 and Q3). For the nulliparous group: min/max range = 19-43; median = 29; Q1 = 24; Q3 = 33. For the parous group: min/max range = 20–44; median = 25; Q1 = 22; Q3 = 38. **b** Example of a healthy mammary gland. Top image: Hematoxylin and Eosin staining showing two-terminal ductular lobular units (TDLU) surrounded by stroma. Bottom images: pre- and post- laser-capture microdissection of the TDLU highlighted in **b**. The section was first stained with an enzymatic reaction, which reacts to the activity of the mitochondrial cytochrome C oxidase and allows easy identification of the epithelial cells. Scale bar = 150 μm. **c**, **d** Total mutation, non-synonym, substitution, and indel burden shown as one dot for each donor, excluding two outliers (donor 25 and 28) reported in Supplementary Fig. 1 (*n* = 27 donors, some dots overlapping). Pearson correlation (*R*) with age, *P* values (*p*) from linear regression and 95% confidence band (grey shade) are shown for the (**c**) epithelial and (**d**) stromal compartment. **e** Distribution of the variant allele frequency (VAF) of the mutations detected in the matching epithelium (Ep, grey) - stroma (St, pink) samples. Kernel density estimation and the average of the median VAF of the samples (dashed lines) is displayed. Distribution is shown for samples carrying a minimum of 3 mutations.

We analyzed a total of 29 frozen healthy breast tissues from donors with no previous use of hormonal contraception and without pathogenic mutations in *BRCA1* or *BRCA2*. In the parous group, uniparous women were preferentially included in the study (*n* uniparous = 15/17) and the average BMI of 29.3 was comparable with the US female population^20^ (Fig. 1a, Table 1 and Supplementary Data 1 for summary and complete clinical information, respectively). The above sample selection criteria were chosen to minimize confounding factors, such as exogenous hormones or multiparity. To represent length of the reproductive years, donors were divided into the following groups: 3 parous and 3 nulliparous <25-year-old; 5 parous and 3 nulliparous between 25–34-year-old; 5 parous and 6 nulliparous between 35–55-year-old. For these parous donors, tissues were collected less than 10 years after pregnancy. Four extra parous samples of older women (ages 57–76) represented samples where the tissue was collected more than 10 years after pregnancy. To increase the detection of mutated cells, present at low frequency in the samples, and to avoid cross-contamination of the two cellular compartments, we laser-captured both stroma and epithelium individually (Fig. 1b). The median sequencing depth was 40.9× and all samples had a depth of >15×, with 90% of the samples characterized by a depth of >30× (Supplementary Data 2).

**Table 1:** Sample information

Donor ID	Ep ID	St ID	Age at collection	Parity	Age at FFTP
Donor 17	a33	b34	23	Parous	21
Donor 18	c35	d36	23	Nulliparous	NA
Donor 4	G7	H8	23	Parous	20
Donor 11	U21	V22	23	Nulliparous	NA
Donor 2	C3	D4	24	Nulliparous	NA
Donor 8	O15	P16	24	Parous	18
Donor 6	K11	L12	28	Parous	20
Donor 7	M13	N14	28	Parous	23
Donor 9	Q17	R18	28	Nulliparous	NA
Donor 14	AA27	BB28	29	Parous	23
Donor 10	S19	T20	29	Parous	23
Donor 13	Y25	Z26	29	Nulliparous	NA
Donor 15	CC29	DD30	32	Parous	27
Donor 12	W23	X24	32	Nulliparous	NA
Donor 16	EE31	FF32	35	Parous	33
Donor 20	g39	h40	37	Nulliparous	NA
Donor 21	E41	S42	38	Parous	32
Donor 3	E5	F6	38	Nulliparous	NA
Donor 5	I9	J10	38	Parous	35
Donor 1	A1	B2	39	Nulliparous	NA
Donor 19	e37	f38	39	Parous	36
Donor 22	E59	S60	45	Parous	39
Donor 25	E65	S66	45	Nulliparous	NA
Donor 28	E75	S76	51	Nulliparous	NA
Donor 29	E77	S78	53	Nulliparous	NA
Donor 27	E71	S72	57	Parous	37
Donor 23	E61	S62	58	Parous	41
Donor 24	E63	S64	63	Parous	23
Donor 26	E67	S68	76	Parous	22

Each donor comprises of an epithelial sample (Ep ID) and a stromal sample (St ID). Age of FFTP indicates age of the donor at first full-term pregnancy. Samples are ordered by age of tissue collection/donation.

### Increase in the mutational burden of the mammary epithelium with age

The mutational burden of the healthy mammary gland highly varied amongst samples, with a median of 19 (range 0–5292) substitutions and 4 (range 0–438) indels in the epithelium and a median of 312 (range 0–1081) substitutions and 32 (range 0–133) indels in the stroma (Fig. 1c, d and Supplementary Data 3). Amongst the epithelial group, we observed the presence of two hyper-mutated samples: donor 25 and donor 28 (Supplementary Fig. 1); once the two donors were removed from the analysis, in the remaining 27 donors, we observed a significant positive correlation between mutational burden and age in the epithelial samples (*n* = 27, *R* = 0.53, *p* = 0.004), while the stromal counterpart showed higher variability, possibly due to a more heterogeneous cellular composition of the latter compared to the epithelium (*R* = 0.31, *p* = 0.118).

We observed no correlation between mutational burden and sequencing coverage amongst the 27 samples, and the mutation spectrum did not differ between the cellular compartments. Comparison of the mutational spectra across epithelium and stroma revealed that the healthy mammary gland contained a predominance of C > T and T > C substitutions. The fitting of known mutational signatures was possible only in 3 epithelial samples with enough mutations (>500), from which we observed two main single-base-substitution signatures, SBS5 and, to a smaller extent, SBS1 (Supplementary Fig. 1c). The presence of SBS5 (unknown etiology) and SBS1 (due to deamination of 5-methylcytosine to thymine), which are both associated with age of the individual in normal and cancer tissues, including BC^21,22^, was also confirmed in the remaining stromal and epithelial samples combined (Supplementary Fig. 1d).

Our data, derived from the DNA of the entire epithelium present in one single biopsy and with germline variants filtered by the stromal counterpart, show a mutational burden comparable with other LCM-based multi-sampling sequencing of other normal tissues, including normal epithelia of female organs^8^.

While our mono-sampling approach limits any phylogenetic reconstruction of the normal tissue, the mean variant allele frequency (VAF) of the mutated cells provides evidence of the presence or absence of a stem cell compartment for the two cellular components of the mammary gland. Both epithelium and stroma of the total 29 donors were characterized by a median VAF of approximately 0.26 with few clonal mutations, suggesting that the normal breast is characterized by a predominant subclonal architecture (Fig. 1f and Supplementary Fig. 2). No difference in the median VAF was observed in the hypermutated samples.

We did not detect copy number changes in most samples, and the 4 detected variants were of length size less than 1 Mb (Supplementary Data 4). However, out of the 2 samples with CNVs, we found amplifications of *AGO2* in one sample, a component of microRNA biogenesis previously reported as amplified in BC samples^23^.

### Mutational burden within breast cancer-associated genes

In the recent years, whole-genome sequences of 560 BC samples allowed the identification of 93 cancer-associated genes carrying both passenger and driver mutations^24^.

**Fig. 2:**
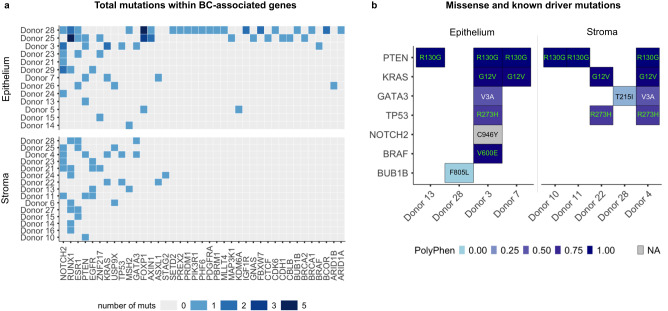
Presence of mutations in breast cancer-associated genes in the healthy breast. **a** Number of mutations (passengers and known driver events) within known BC-associated genes within the epithelium and stroma of healthy individuals (total of 29 donors). For each donor, mutations are unique to one cellular compartment. **b** Representation of the subset of identified missense mutations, including of known pathogenic variants curated by OncoKB™ (highlighted in green), with respective PolyPhen score (0 = benign, 1 = damaging) in each sample in the epithelium and stroma of healthy donors. The change in amino acid is reported within each tile.

We detected somatic mutations in 38/93 and 13/93 of the above identified genes in the epithelium and stroma, respectively, albeit at different frequencies (Supplementary Data 5 and Fig. 2a). This is in line with findings from other healthy tissue studies which confirm the presence of mutations in cancer-associated genes in the normal epithelium and blood^3,4,6–8,10,25^. As expected, the higher number of mutations in the hypermutated donors 25 and 28 corresponded to a higher number of mutations in cancer-associated genes. In the breast, there was a preferential accumulation of mutations in *NOTCH2* in the epithelium and *RUNX1* in the stroma, mutated in approximately 21% (6/29) and 24% (7/29) of samples in the two cellular compartments, respectively (Fig. 2a). Within these two genes, the majority of the identified mutations were of non-coding nature. *NOTCH2* carried a total of 7 unique mutations in 9 donors: a novel missense mutation (p.C946Y) detected in 1 epithelium sample; a mutation within the 5’ UTR region (rs79247096) detected in 2 epithelial samples; a silent mutation (rs139358772, p.P765 =)  present in both stroma (2 donors) and epithelium (2 donors); 4 mutations in introns, including one (rs386635149) present in 3 donors (1 epithelium and 2 stroma occurrences).

RUNX1 carried a total of 11 unique mutations in 10 donors. The majority of these (10/11) were present within introns, including one (rs71326607) present in 4 donors (1 epithelium and 3 stroma occurrences). One mutation within the 5’ UTR region (rs756296267) was detected in 1 epithelial sample.

From our data, we also identified the presence of previously reported BC driver mutations^24^, in oncogenic mutations found in the OncoKB database^26^, and missense mutations with possible clinical implication, measured with Polyphen score.

The missense mutations in *PTEN* resulting in p.R130Q or p.R130* are recognized driver events in BC^24^. We recorded a different variant of this mutation in the same genomic position, resulting in p.R130G, a pathogenic variant (PolyPhen score = probably damaging, 0.999) typically associated with endometrioid carcinoma, amongst other pathologies^21^. This mutation was found at an unexpected high frequency in the healthy breast, in the epithelium of 3/25 individuals (~10%), and in the stroma of unmatched 3/25 individuals (~10%). Interestingly, this mutation is nearly clonal (VAF > 0.70) in one epithelial and one stromal sample, independently (Supplementary Data 5 and Fig. 2b).

Known driver events in *KRAS* (pG12V) and *TP53* (p.R273H) were also found in both distinct epithelial and stromal compartment in 4 and 3 donors, respectively, some with VAF > 0.8 (Supplementary Data 5 and Fig. 2b). Finally, we identified missense mutations deemed probably or possibly damaging by PolyPhen in *GATA3* (p.V3A), *BRAF* (p.V600E), two benign mutations in *BUB1B* (p.F805L) and *GATA3* (p.T215I), and a novel missense mutation of unknown impact in *NOTCH2* (p.C946Y) (Supplementary Data 5 and Fig. 2b).

### Parity affects the age-dependent mutational burden of the healthy breast

Epidemiologic and molecular studies have determined a double role of pregnancy in the contribution of BC risk. Women who reach full-term pregnancy before the age of 24 have a lower long-term BC risk compared to age-matched nulliparous women^15,17^. On the other hand, women who give birth for the first time after age 35 are more likely to develop BC than early-pregnancy age-matched groups^15^. First-age of pregnancy exerts a protective role only towards ER + BC^27,28^, but multiparity has not been uniquely associated with protection towards BC carrying specific hormonal status^27,29^. For both age groups, short-term BC risk significantly increases after each pregnancy, seen in the subsets of BC that occurs during pregnancy (PrBC) and during the postpartum period (PPBC)^30–34^. While both PrBC and PPBC are associated with higher mortality and recurrency rates compared to non-pregnancy-related BC, amongst the latter, the risk of developing ER- BC is highest 2.2 yeas after birth, and post-weaning BC is characterised by a worse prognosis^35–38^. Therefore, our next aim was to detect the combined contribution of both age and parity to the mutational burden in the healthy breast. Here, we defined parity as the phase of tissue remodeling, which commences at conception and has an effect up to 10 years after pregnancy^14^. For this analysis, we, therefore, excluded the 4 parous donors from which more than 10 years have passed between the last pregnancy and tissue collection, a time during which the direct effect of parity is not easily determinable. We also excluded the two hypermutated samples which were both from nulliparous donors (final n of analyzed donors = 23). The latter are shown in Supplementary Fig. 3.

**Fig. 3:**
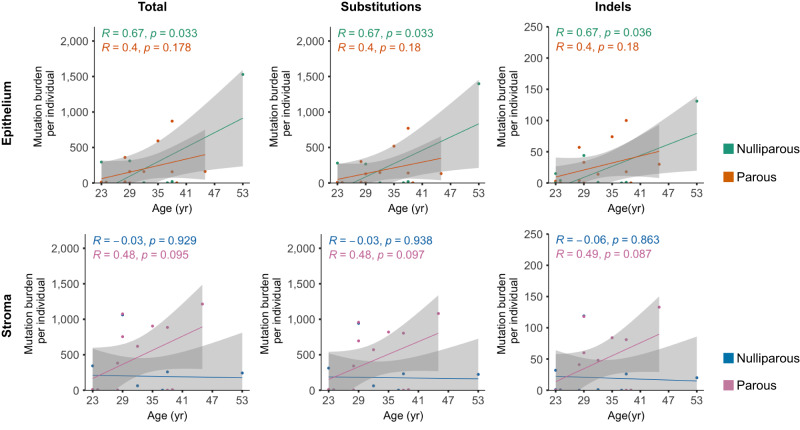
Effect of parity to the mutational burden of the healthy breast. Correlation between mutational burden (total burden, and burdens of individual substitutions, and indels) with and age of tissue collection, for the (upper panel) epithelial and (lower panel) stromal compartment. Data shown as one dot for each donor, with the two nulliparous hypermutators and the 4 older parous donors excluded (*n* = 10 nulliparous, *n* = 13 parous showing, some dots overlapping). Pearson correlation (*R*) with age, *P* values (*p*) from linear regression and 95% confidence band (grey shade) are shown.

In the analyzed nulliparous epithelial samples (*n* = 10), the number of substitutions (single, double, and triple nucleotide changes) significantly increased with age (*R* = 0.67, *p* = 0.033), a tendency which was weaker and did not reach statistical significance in the parous epithelium (*n* = 13, *R* = 0.40, *p* = 0.178, Fig. 3). The initial observed mutational burden independently of parity allowed an estimate of approximately 15.24 ± 5 mutations/year (muts/year) in the epithelium (Fig. 1c).

In the same individuals, compared to the epithelium, the stromal compartment accumulated mutations with an opposite trend. Here, the parous stroma accumulated mutations with age, albeit not significantly (*R* = 0.48, *p* = 0.095), but the nulliparous stroma did not show the same trend (*R* = −0.03, *p* = 0.929, Fig. 3). We could estimate a total accumulation of approximately 9 ± 6 muts/year in the stroma (Fig. 1d). In the parous stroma, the rate amounts to approximately 42 ± 17 muts/year.

### Parity affects the age-dependent median variant allele frequency of the mammary gland

**Fig. 4:**
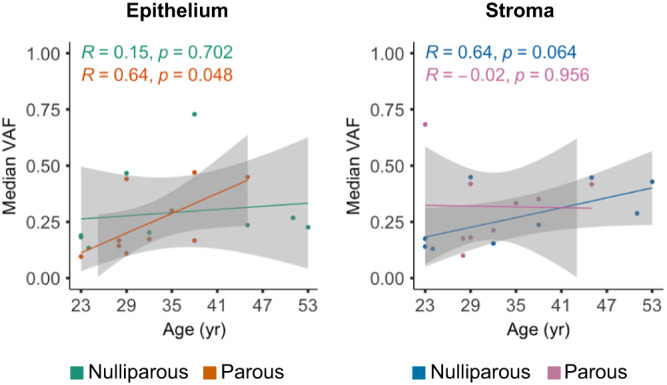
Effect of parity to variant allele frequency of the healthy breast. Correlation between the median VAF of all the mutations with and age of tissue collection, for the epithelial and stromal compartment. In both graphs, data was restricted to the 21 donors which were characterized by a total of >15 mutations in the two cellular compartment. Parous donors whose childbirth occurred >10 years before tissue collection were also excluded (*n* = 9 nulliparous, *n* = 10 parous showing, some dots overlapping). Pearson correlation (*R*) with age, *P* values (*p*) from linear regression and 95% confidence band (grey shade) are shown.

Our monosampling does not permit to perform a phylogenetic analysis of the clonal evolution in the mammary gland, but the allele frequency can still provide information on how mutations expand in the tissue. To determine the effect of parity on the clonal expansion of the somatic mutations, we have restricted our analysis to 21 donors which were characterized by carrying a total mutational burden of >10 mutations within the two cellular compartments. As above, the 4 parous donors with a later childbirth after tissue collection were also excluded. Only in the parous epithelial cohort (*n* = 10), age was positively correlated with an increase in the median VAF of mutations (*R* = 0.64, *p* = 0.048, Fig. 4, orange line). On the other hand, the median VAF of the mutations is not altered in older epithelial nulliparous donors compared to the younger cohort (*n* = 9, *R* = 0.15, *p* = 0.702, green line). Interestingly, in the same donors, the mammary stroma showed a different trend, where an increase in age was positively correlated with an increase of the median VAF, albeit not significantly, in the nulliparous donors (*R* = 0.64, *p* = 0.064, Fig. 4, blue line), but not in the parous donors (*R* = −0.02, *p* = 0.956, Fig. 4, pink line).

Taken together, these data suggests that the fast proliferation and differentiation characteristic of the mammary epithelium during pregnancy induces expansion of pre-existing clones, rather than the onset of new mutations. On the other hand, single cell sequencing strategies may be required to understand the cellular dynamics of the heterogenous stroma, which could explain which type of stromal cells are the cause of the difference in the observed clonal expansion compared to the mammary epithelium.

## Discussion

In recent years characterization of the mutational and clonal dynamics of several healthy tissues has provided insight into the architecture of normal epithelia and shown evidence of cancer-associated mutations before the onset of the disease.

Due to technical and physiologic challenges of sequencing the mammary epithelium in its native state (without amplification of the cells in vitro), the mutational landscape of the healthy breast is largely uncharacterized, even though a recent study on RNA sequences reveals clonal expansion in the mammary gland^39^. Even less information is known regarding the genetic composition of mammary stroma, a heterogeneous cellular compartment that plays a crucial role during involution and in inflammatory processes of the breast, including inflammatory BC^40^. Finally, the interplay between parity and age at first pregnancy is one of the most complex factors in BC risk, but their individual and combined contributions to the accumulation of somatic mutations in the healthy breast have not been investigated.

Our results show that, in line with other healthy organs^1–5,7–10^, the healthy breast significantly accumulates mutations with age, the significance of which is evident in the epithelial compartment. Compared with published data derived from WGS of bulk BC^24^, our data show a minimum of a 3-fold decrease in the number of substitutions in the healthy breast; however, it is important to highlight that the differences in sequencing method do not allow an exact comparison between the datasets. The rate of accumulation in the nulliparous breast is lower than other tissues, including the endometrium, and mutated clones are maintained at a constant size with age. This can be expected from a tissue for which the cellular renewal rate is maintained constant during the adult nulliparous life, until menopause^12,41^. While it has been in fact shown that mammary cell proliferation increases during the luteal phase of the menstrual cycles, possibly due to progesterone-induced paracrine signaling, cellular homeostasis is maintained throughout the menstrual cycles by the fluctuating apoptosis during early follicular and late luteal phase^12,41,42^ and reviewed by Brisken et al.^43^.

**Fig. 5:**
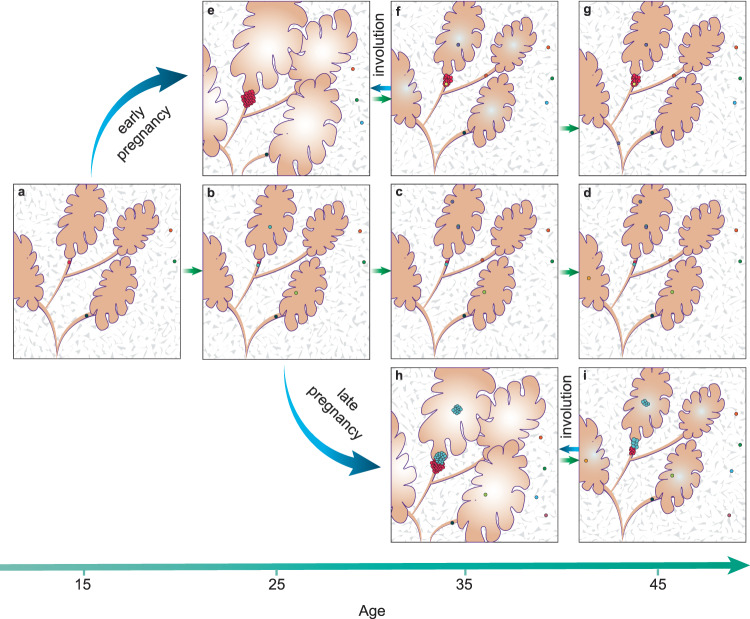
Model of expansion of a single mutated clone in the healthy breast. The scheme summarizes a model on how the number of mutations (circles) and clone size vary during aging in the parous and nulliparous epithelium (pink) and stroma (grey). Pregnancy-associated clone expansions or regression are represented by blue arrows; age-related clone expansion is represented by green arrows. In the event of life-long nulliparity (**a**–**d**), the number of mutations increases in the epithelium, but not in the stroma. In the event of early pregnancy (**e**–**g**), the epithelial clone undergoes significant expansion, followed by shrinkage due to post-partum involution (**f**) and depending on the mutation fitness. Similar clone expansion and shrinkage occur after a late pregnancy, but the number of bigger mutated clones is higher than in individuals of the same age from the nulliparous or early parous group, due to the contribution of age-related accumulation of mutations to pregnancy (**h**, **i**).

On the other hand, we showed that modulation from pregnancy to the architecture of the mammary gland is a complex phenomenon, also affected by the age of the individual which could in part explain differences in BC risk. Our mutational analysis however cannot directly explain the higher risk in the onset of estrogen receptor positive BC amongst older mothers. The regulation of the expression of estrogen receptor in human cancer is complex with factors responsible including epigenetic regulation, alternative splicing, and posttranscriptional mechanisms^44^. An elegant study using mice intraductally injected with RCAS-caErbb2 demonstrated how a full-term pregnancy accelerates oncogenesis by promoting the growth of early lesions from cells carrying a previously activated ErbB2^45^. In a similar way, our data leads us to hypothesize that cell clones containing a pre-existing mutation (such as those in a driver gene) in the nulliparous breast may undergo a rapid expansion with pregnancy, at a probability that is proportional to the fitness of the mutation^46^. As the number of mutations also increases with age before pregnancy, the probability of gaining a second driver mutation within the same clone would be higher in an older parous woman compared to a younger parous or nulliparous individual, who is characterized by a lower mutational burden (Fig. 5). The relatively low mutational burden of the parous epithelium fits with Russos’s concept of breast differentiation, which indicates a higher proliferation of the developing nulliparous epithelium compared to the differentiating pregnant epithelium, where proliferation is lower^47^.

To confirm and complete this model, further studies are required, in particular, to determine the role of menarche, involution, and menopause, in the accumulation of mutations and clonal dynamics, incorporating the use of recently published validated techniques^48^.

The impact of the onset of both menarche and menopause, which has been linked to different rates of BC, is still very much unknown^49^, while several studies have determined the contribution of involution to the mammary architecture. Recent studies have also identified genes differentially expressed in the healthy breast, and in particular in the cells shed by lactating milk, which are mostly epithelial and immune cells^50^. Involution is characterized by a strong apoptotic drive, which restores the epithelium to pre-pregnancy status. In a similar way to observations in mice where it has been hypothesized that pre-existing premalignant mammary cells may be resistant to involution-related apoptosis^45^, we hypothesize here that a minority of clones carrying mutations of higher fitness may be resistant to clone shrinkage during involution, which could explain the transient risk of developing BC after childbirth^32,37,51^.

From our data, we observed a trend of accumulation of somatic mutations in the older parous stroma. While the correlation is not statistically significative, the evident trend may provide some insights into the possible stromal dynamics which occur during pregnancy and involution, and during age-related lobular involution (LI).

In healthy nulliparous women, during LI, the intralobular stroma becomes enriched in connective tissues, while adipocytes considerably increase within the interlobular region, giving rise to a lower density tissue^52^. On the other hand, during pregnancy, a different level of reorganization occurs to allow the development of the pregnant epithelium. The entirety of the stromal cell types are affected by changes in angiogenesis, immune infiltration, loss of lipid accumulation within adipocytes, independently of age (reviewed by McCready et al.^53^). After weaning, the developmental changes induced by pregnancy reverse to a pre-pregnancy state in a process known as post-lactational involution. In the stroma, an influx of immune cells clears the debris of the apoptotic epithelium, fibrillar collagen deposition and proteolysis of collagen occur in the extracellular matrix, lymphangiogenesis ensues, and adipocytes undergo differentiation to allow again lipid accumulation (ref. ^54^ and reviewed^53,55^).

We therefore speculate that the post-lactational stroma, which is represented by our parous cohort, is in a unique position where age-related and post-pregnancy involution occur at the same time. In this scenario, it is possible that observed increase in somatic mutations in the aging parous stroma is associated with more evident changes in the cellular dynamics, as the mammary stroma underwent more intense modifications to first exit a state of LI (which can start several years before menopause) to allow pregnancy and then involution. We also consider that these differences are also reflected in differences epigenetic changes and gene expression profiles between specific cell types within the younger and older parous groups. Studies of phenotypic and genotypic heterogeneity of the stroma during age and pregnancy performed with single-cell analysis may possibly provide further insights into the interplay between epithelium and stroma of the mammary gland.

It is essential to recognize the limitations of the study, especially mono-sampling and a moderate number of patients. While previous studies have shown how breast tissues from the Komen Tissue Bank are the ideal source of material for this type of research, as they are characterized by less histologic abnormalities than reduction mammoplasty specimens, which reflect in a lower cancer risk within the cohort^56^, the amount of available tissue is limited and does not allow the analysis of multiple biopsies. On the other hand, while sampling the epithelium or stroma as a bulk allows the detection of several somatic mutations comparable to other published studies, it may not be sensitive enough to detect small clones, especially in driver genes.

Our approach presents however several advantages specific to working with the mammary gland. The use of breast tissue from healthy donors rather than from reduction mammoplasty or tissue adjacent to cancer avoids bias due to differences in BMI and contamination with cancer-associated changes occurring in the adjacent epithelia^39^. The direct comparison between laser-captured epithelium and stroma allows an increase in specificity and comparable sensitivity to the use of blood or other non-mammary tissues as controls for germline mutations and allows the characterization of the genetic changes in the often-neglected stromal compartment. On the other hand, the inclusion of controls from blood or from bulk tissues and the implementation of recently published guidelines for the sequencing of low-input DNA would ensure a reduction of false positive variants^48^. In this study, we utilized MuTect2 as our variant calling tool of choice, accompanied by a series of filters to increase the confidence in observed somatic variants. Despite its effectiveness, like any variant caller, MuTect2 can produce false positive results for somatic mutations due to inadequate filtering of sequencing noise, particularly when a limited number of samples are available to generate a panel of normals. Benchmarking the performance of the caller with the chosen sequencing technology, alongside the analysis of mutational signatures, which demonstrated a lack of sequencing artifacts, underscored the efficacy of additional filters in mitigating the impact of potential false positives.

As a result, we were further able to determine the presence of mutations within BC-associated genes in a subset of samples, indicating that early pathogenic events in the healthy tissue could expand with time and give rise to cancer, similar to what occurs in other healthy organs. Intriguingly, our data revealed an accumulation of mutations in *NOTCH2* and *RUNX1* in the healthy breast, independently of parity. The Notch family of transmembrane proteins has a role in the establishment of the basal and luminal epithelial lineages during normal breast development^57^. In particular, Notch2 can regulate Notch1 and Notch3^58^ and has been shown to be involved in controlling branching morphogenesis^59^. *NOTCH2* mutations are present in BC, in up to 26% of cases^60^, but the occurrences found in this study have not previously implicated in BC pathogenesis. Deeper functional studies are therefore required to reveal the potential functional meaning of the mutations in breast cells.

Most mutations found in *RUNX1* were located within introns and their significance is unknown. However, we identified an interesting common intronic mutation (rs71326607) present in more than one donor. By default, the annotations detected by Ensembl VEP use the Ensembl/GENCODE transcript as a reference, rather than NCBI’s RefSeq transcripts. Analyzing the latter, we observed that the common mutation resides within an uncharacterized LOC101928269, corresponding to the validated ncRNA NR_110418.1, which is not automatically annotated by VEP. The functions of this ncRNA have not been elucidated yet, and our finding may offer an avenue for further research. We have also identified 4 individuals characterized by the unusual presence of more than one non-synonymous mutation at high frequency, but we did not record any difference between these individuals and the remaining samples in terms of sequencing depth, amount of collected cells, or family history. A multi-sampling approach would be particularly useful to understand the clonal dynamics of these individuals. The same approach would also allow us to determine which genes undergo positive selection in the healthy breast. However, to perform a confident estimate of the dN/dS ratio, a multi-sampling approach cohort^61^, or a deeper sequencing methods, are needed, which is problematic when working with low amount of epithelial cells characteristic of this cohort^56^.

In aggregate, the mutational landscape of the healthy breast can be used as a control for the genetic study of diseases of the mammary gland, including non-malignant lesions. In BC studies, there is a need for a real “pool of normal” controls, rather than controls derived from areas adjacent to or surrounding cancer, or from the contralateral breast, both of which have been used until now for this scope. These controls, collected from affected patients, may, in fact, include, albeit at low frequency, mutations that are derived from cancer^62^. Furthermore, although BC generates from the epithelial cells of the duct, sequencing of the stromal compartment, which has not been undertaken before, may provide some insight into the elusive onset of inflammatory breast cancer, a subset of BC characterized by the dysregulation of several major inflammatory signaling pathways^63^.

## Methods

### Human breast samples

Twenty-nine frozen normal breast tissue samples, constituted by one biopsy per donor, from patients not affected by cancer were obtained from the Susan G. Komen Tissue Bank at IU Simon Cancer Center, following patient consent and approval from the local research ethics committee (IRB Protocol number is 1011003097). Human samples used in this research project were stored in the Imperial College Healthcare Tissue Bank (ICHTB) with sub-collection ONC_JS_18_001. ICHTB is supported by the National Institute for Health Research (NIHR) Biomedical Research Centre based at Imperial College Healthcare NHS Trust and Imperial College London. ICHTB is approved by Wales REC3 to release human material for research (REC 17/WA/0161). This work was performed following the ethical principles in the Declaration of Helsinki. See Supplementary Data 1 for clinical information.

To confirm the normal histology, hematoxylin and eosin (H&E) staining was performed on the first section of each sample. Each section was cut at 5μm thickness, hydrated in decreasing concentration of ethanol, and stained using Gills Hematoxylin and 1% Eosin, as per standard protocol.

### Enzyme histochemistry

To allow visualization of the epithelium, enzyme histochemistry targeted at a mitochondrial enzyme was performed on the section. Cytochrome C oxidase (CCO) enzyme histochemistry was performed on 16 μm thick serial sections cut onto PALM membrane slides (MembraneSlide 0.17 PEN, Zeiss, Munich, Germany), as previously described^64^. Briefly, thawed sections were incubated in cytochrome c medium (100 mM cytochrome c, 4 mM diaminobenzidine tetrahydrochloride, 20 μg/ml catalase in 0.2 M phosphate buffer, pH 7.0; all from Sigma Aldrich, Poole, UK) for approximately 50 min at 37 °C or until satisfactory visualization of the brown color, followed by washes in PBS, pH7.4, for 3 × 5 min. Sections were allowed to air-dry before proceeding to microdissection. CCO-wild type cells, present in the majority of the normal epithelium, are visible in brown, in contrast with the surrounding colorless stroma.

### Laser-capture microdissection and DNA extraction

Stained areas from the mammary epithelium, containing mammary ducts and terminal duct lobular units (TDLUs), were microdissected on a laser capture system (Zeiss PALM Microbeam) at a uniform laser power and cutting width. Approximately 30 sections were dissected for each individual. Surrounding stromal tissue with absence of detectable epithelium was superficially cut with the laser and successively isolated under a macrodissecting microscope (Leica Microsystem, Germany). Dissection and DNA extraction of the stroma and epithelium of each individual sample was performed at separate times to avoid contamination.

Pre and post dissection images were captured using a digital slide scanner and viewed using the integrated viewer software (NanoZoomer SQ, Hamamatsu Photonic, Shizuoka, Japan).

DNA was extracted using QIAamp DNA Micro kits (Qiagen, Hilden, Germany) and measured on Qubit 3.0 Fluorimeter, using Qubit dsDNA HS Assay kit (Life Technologies, Paisley, UK), according to the manufacturer’s recommendation.

### Library preparation and DNA sequencing

Library preparation and sequencing were carried out by the Beijing Genomic Institute (Shenzhen, Guangdong, China). In brief, isolated DNA (100 ng) was fragmented by Covaris technology to obtain fragments of an average 350 bp length. The repaired/dA-tailed DNA fragments were then ligated to both ends with the adapters and amplified by ligation-mediated PCR (LM- PCR), followed by single-strand separation and cyclization. A rolling circle amplification (RCA) was performed to produce DNA Nanoballs (DNBs). The qualified DNBs were loaded into patterned nanoarrays and 100 bp pair-end read sequences were read on the BGISEQ-500 platform, which studies have shown to be of comparable sensitivity to other commercial platforms^65^. Sequencing-derived raw image files were processed by BGISEQ-500 base-calling software (Zebracall process version 0.5.0.13875) with default parameters and the sequence data of each individual is generated as paired-end reads.

### Mutations calling and downstream analysis

Alignment of the reads and calling were carried out by the bioinformatics team at the Beijing Genomic Institute, following the guidelines of the Broad Institute Genome Analysis Toolkit (GATK, https://www.broadinstitute.org/gatk/guide/best-practices). Briefly, cleaned data for each sample were mapped to the human reference genome (GRCh37/HG19) with Burrows-Wheeler Aligner (v0.7.12), and duplicated reads were removed by Picard-tools (v1.118) with default settings. Details of quality control, coverage, and mapping can be found in Supplementary Data 2.

Somatic short mutations, including SNV, small insertions, and deletions, were called via local assembly of haplotypes with Mutect2 (v4.1.4.1, https://gatk.broadinstitute.org/hc/en-us/sections/360007458971-4-1-4-1), the performance of which was previously established using synthetic whole-genome sequencing^66^, with an adaptation of the “tumor with matched normal” function. To report both stroma- and epithelium-only mutations, the analysis therefore run as “epithelium vs stroma” and “stroma vs epithelium”. Default settings for tumor-normal pairs were used. Called variants were successively filtered with FilterMutectCalls (v4.1.4.1, https://gatk.broadinstitute.org/hc/en-us/sections/360007458971-4-1-4-1), and only mutations categorized with PASS, representing confidently called somatic mutations, were used in the downstream analysis. VCF files were converted to Mutation Annotation Format (MAF) files according to National Cancer Institute specifications using Mskcc/vcf2maf (v1.6.17)^67^, adapting the format to include epithelial and stromal barcodes instead of tumor and normal barcodes, respectively.

Annotated mutations were then further filtered for (a) the total number of reads > 10; the mutated number of reads > 5; variant allele frequency (VAF) > 0.02%; no reads in the control compartment; (b) removal of artifacts stemming from sex chromosome homology and repetitive regions (RepeatMasker 4.1.1, http://www.repeatmasker.org/); (c) frequency > 0.1% in the Genome Aggregation Database (gnomAD, V2.1.1, exome and genome samples, and gnomAD V3.1 https://gnomad.broadinstitute.org/), in the 1000 Genome database (https://www.internationalgenome.org/) and ALFA: Allele Frequency Aggregator (https://www.ncbi.nlm.nih.gov/snp/docs/gsr/alfa/ALFA_20200227123210); (d) removal of possible germline mutations not detected with FilterMutectCalls present in a minimum of 2 samples from the pooled panel of matching compartment (epithelium or stroma).

To ensure the minimal occurrence of false positives derived from either the presence of common polymorphisms or from the sequencing technology, thus increasing the accuracy of somatic variant calling in BGI-sequenced data using Mutect2, our calling pipeline was further benchmarked on a BGI-sequenced sample (NA12878) deposited in the Genome In A Bottle (GIAB) consortium database using the “platinum” truth variant catalogue previously generated^68^, filtered to obtain only somatic calls (Supplementary Methods and Supplementary Fig. 4).

MAF were visualized and summarized using the R Bioconductor package, maftools (v1.8.10)^69^ when necessary before proceeding to detailed analysis.

Variants were categorized into synonymous and non-synonymous according to the impact on protein-coding of each variant (Ensembl IMPACT rating). Non-synonymous variants with high or moderate IMPACT rating were: Frameshift deletions, Frameshift insertions, Nonsense mutations, Nonstop mutations, Splice sites, Translation start sites, In-frame deletions, In-frame insertions, Missense mutations. Synonymous mutations with low or modifier IMPACT were: 3’ Flank, 5’ Flank, 3’ UTR, 5’ UTR, Intron, IGR, Splice regions, RNA, Silent mutations.

Downstream analysis was conducted in R^70^ and all figures were produced using the package ggplot2 (v3.3.0)^71^, unless otherwise specified. Linear regression analysis provided estimates for the change of the mutational burden with age.

### Determination of mutations occurring in breast cancer-associated genes

The list of identified breast cancer (BC) drivers and individual driver events included in our analysis were taken from the paper “Landscape of somatic mutations in 560 BC whole-genome sequences” by Nik-Zainal et al., Nature, 2016^24^. Annotations on the oncogenic effects of the proteins were taken from OncoKB™ (version updated in October 2022)^26^.

### Identification of mutational signatures

The contribution of known mutational SBS signatures from the COSMIC database^21^ was determined using the R Bioconductor package MutationalPatterns (v3.0.1)^72^. Briefly, VCF files were imported as *GRanges* object and the sequence context was derived from the imported Reference Genome hg19, installed with the R Bioconductor package BSGenome (v1.58.0)^73^. To avoid overfitting of signatures, the function *fit_to_signatures_strict* was implemented with a final cutoff of 0.005.

### Copy-number variants

Copy-number variants were called by cn.MOPS (v1.44.0)^74^, using the *getReadCountsFromBAM* function with a window length parameter WL = 20000, according to the authors’ recommendations. The function *referencecn.mops* was applied to allow a modified “Tumor vs. Normal” setting, where the tumor was substituted with normal epithelium, and matched normal was substituted with matched normal stroma. Variants were annotated with AnnotSV (v3.1)^75^ with the following settings to call and benign entries: *benignAF* = 0.01%, *minTotalNumber* = 500. Benign variants are automatically included by AnnotSV from the following datasets: ClinVar, ClinGen, Database of Genomic Variants (dgv, nsv or esv), gnomAD, Deciphering Developmental Disorders, 1000 Genomes, Ira M. Hall’s lab, Children’s Mercy Research Institute. The remaining parameters were set to default. AnnotSV also allowed the filtration of putative calls present in problematic regions of the genome (ENCODE blacklist^76^). All putative regions were further manually inspected on Integrative Genomics Viewer (IGV, v2.4.19). The reads were compared to those of the matched samples, and true positive calls were validated on the presence of drops in the coverage compared to both matched control and neighboring regions, split-reads or paired-end abnormal signal, as previously reported^77^.

### Reporting summary

Further information on research design is available in the Nature Portfolio Reporting Summary linked to this article.

### Supplementary information

Supplementary Information

Description of Additional Supplementary Files

Supplementary Data 1-5

Reporting Summary

## Data Availability

All data is available in the main text or Supplementary materials. Aligned reads (BAM format) are available via the European Genome-Phenome Archive with accession number EGAS00001004672. The raw sequencing data are available under restricted access due to data privacy laws; access can be requested to the Data Access Committee as detailed here: https://ega-archive.org/access/data-access. The GRCh37/hg19 version of the human reference genome (http://genome.ucsc.edu) has been employed in this study.
