# Supplementary material for: The mutational landscape of the adult healthy parous and nulliparous human breast
Source: Nat Commun. 2023 Sep 6;14:5136. doi: 10.1038/s41467-023-40608-z (PMC10482899; doi:10.1038/s41467-023-40608-z)
Supplement: Supplementary file 1 — Supplementary Information [file 41467_2023_40608_MOESM1_ESM.pdf]

The mutational landscape of the adult healthy parous and nulliparous human breast

Biancastella Cereser[1,*], Angela Yiu[1], Neha Tabassum[1], Lisa Del Bel Belluz[1], Sladjana Zagorac[1,2], Kenneth Russell Zapanta Ancheta[1,3], Rongrong Zhong[1], Cristian Miere[1], Alicia Rose Jeffries-Jones[1], Nina Moderau[1], Benjamin Werner[4], Justin Stebbing[1,5,*]

[1] Cancer Genetics Group, Department of Surgery and Cancer, Imperial College London, London, United Kingdom

[2] Present address: Molecular Oncology Programme, Growth Factors, Nutrients and Cancer Group, Centro Nacional de Investigaciones Oncológicas, Madrid, Spain

[3] Present address: Pathobiology and Population Sciences, Royal Veterinary College, Hatfield, United Kingdom

[4] Evolutionary Dynamics Group, Centre for Cancer Genomics and Computational Biology, Barts Cancer Institute, Queen Mary University of London, London, United Kingdom

[5] Department of Life Sciences, Anglia Ruskin University (ARU), Cambridge, United Kingdom

[*]Joint corresponding authors:

Biancastella Cereser, b.cereser@imperial.ac.uk

Justin Stebbing, justin.stebbing@aru.ac.uk

SUPPLEMENTARY INFORMATION

Supplementary Figure 1 - Mutational burden of the healthy mammary gland
Supplementary Figure 2 - Variant allele frequency of the healthy mammary gland
Supplementary Figure 3 - Effect of parity on the mutational burden of the healthy breast
Supplementary Figure 4 - Benchmarking Mutect2 in BGI-sequenced data
Supplementary Methods
Supplementary References

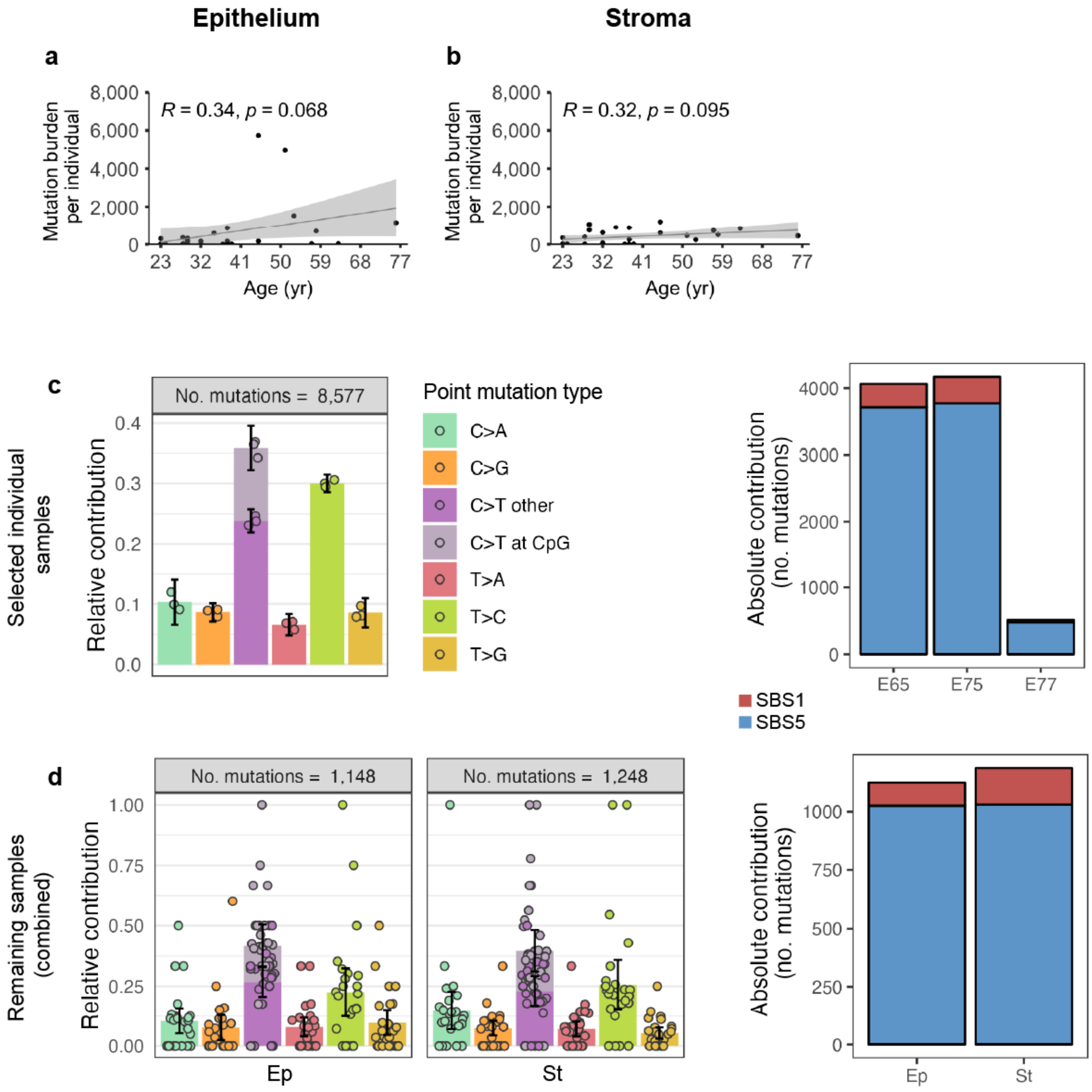

Supplementary Figure 1. Mutational burden of the healthy mammary gland
(**a,b**) Total mutational burden shown as one dot for each donor, including two outliers, samples from donor 25 and donor 28 (n = 29 donors, some dots overlapping). Pearson correlation (*R*) with age, P values (*p*) from linear regression and 95% confidence band (grey shade) are shown for the **(a)** epithelial and **(b)** stromal compartment. (**c-d**) on the left, relative contribution of each mutation type to the point mutation spectrum and on the right, absolute contribution of single-base-substitution signatures found in: **c)** the epithelial subset of samples with >500 SNVs (n=3); **d)** the remaining epithelial (Ep, n=26) and stromal samples (St, n=29), combined. In the graph showing the mutational spectrum, data are represented as the mean relative contribution of each mutation type, with each dot representing one sample and error bars representing the standard deviation.

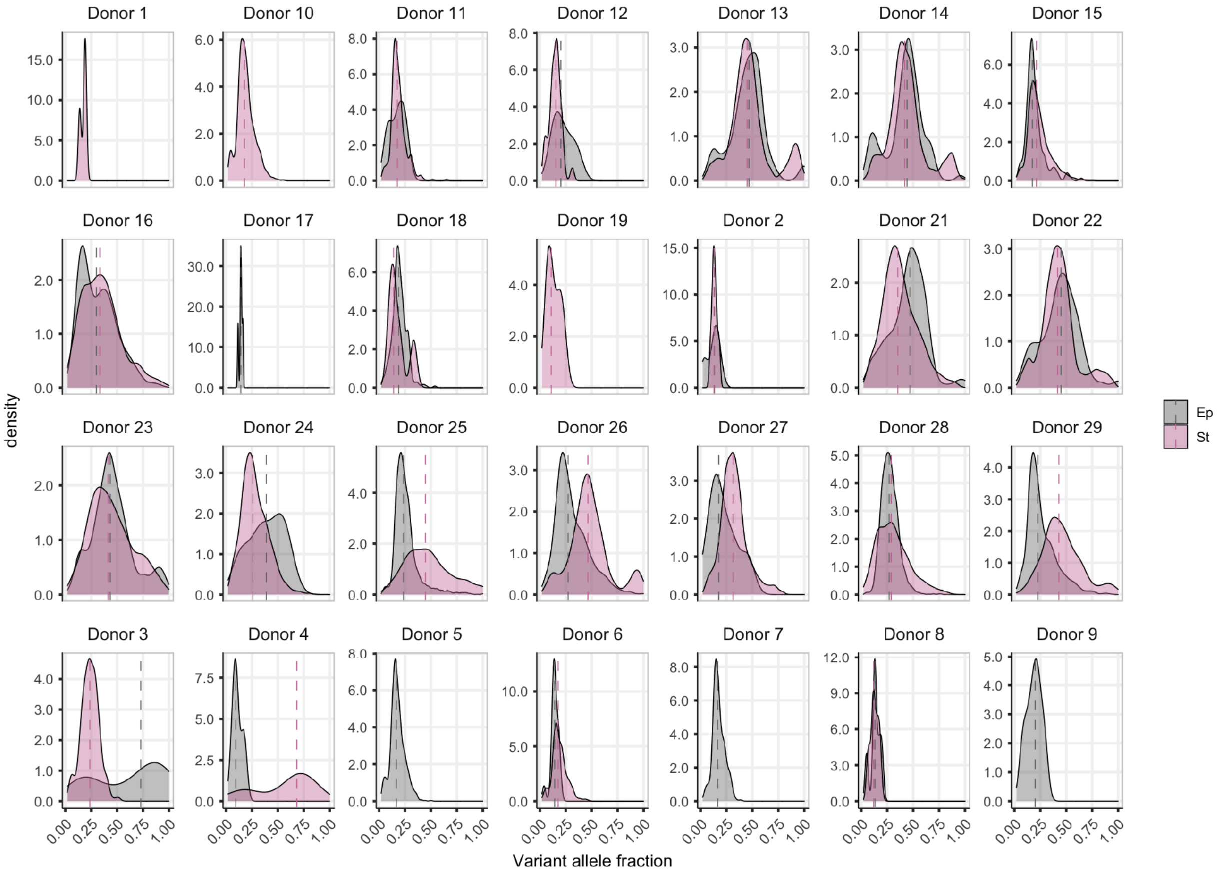

Supplementary Fig. 2 Variant allele frequency of the healthy mammary gland
Distribution of the variant allele frequency (VAF) of the mutations detected in the matching epithelium (Ep, grey) - stroma (St, pink) of individual samples. Kernel density estimation and median VAF (dashed lines) is shown for each sample. Distribution is presented for samples carrying a minimum of 3 mutations. No significant correlation could be found between the median VAF and age of collection of the donor.

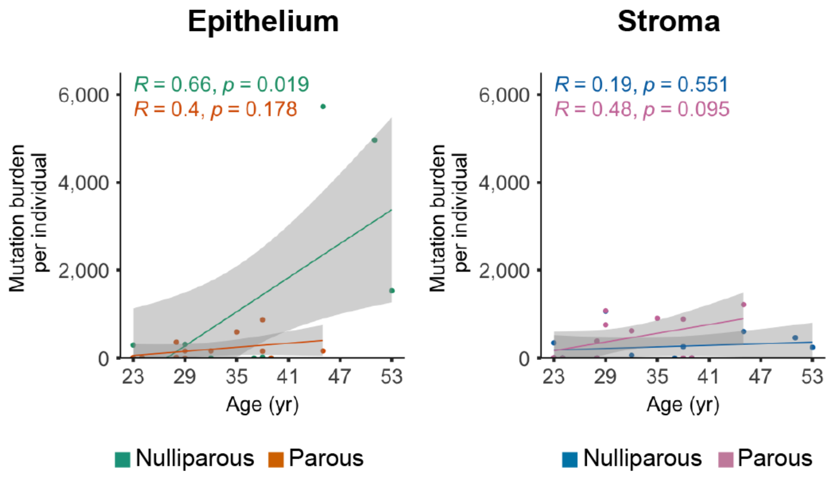

Supplementary Fig. 3. Effect of parity on the mutational burden of the healthy breast
Total mutational burden shown as one dot for each donor (n = 12 nulliparous, n = 13 parous showing, some dots overlapping). The two outliers with an elevated number of mutations, samples from donor 25 and donor 28, are also included. Parous donors who gave birth >10 years before tissue collection were excluded from the analysis. Pearson correlation (*R*) with age, P values (*p*) from linear regression and 95% confidence band (grey shade) are shown.

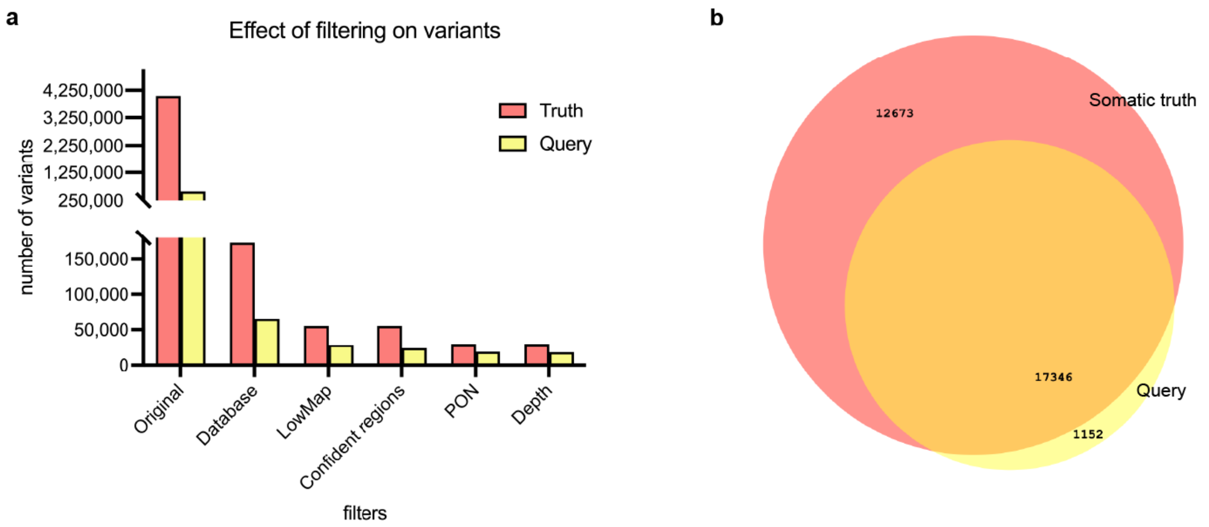

Supplementary Fig. 4. Benchmarking Mutect2 in BGI-sequenced data
a) The bar chart illustrates the reduction in the number of variants after each filter for both the truth dataset and the Mutect2-generated query. A reduction of approximately 99% in the number of calls in the truth dataset was obtained through our filtration process, which likely correspond to the number of germline mutations present in the original file. b) The Venn diagram indicates the number of true positives (calls present in the truth somatic file and assigned somatic by Mutect2), false positives (calls absent in the truth somatic file but assigned as somatic by Mutect2), and false negatives (calls present in the truth somatic file but not assigned as somatic by Mutect2).

SUPPLEMENTARY METHODS

Filtering and benchmarking somatic variants in BGI-sequenced data using Mutect2

In this section, we describe the methods employed to filter and benchmark somatic variants in BGI-sequenced data using the Mutect2 somatic caller. The primary aim was to evaluate the number of false positives (FP) in the context of the "Platinum" dataset (referred to as "truth dataset" below), which includes both germline and somatic variants and was developed by Eberle and collaborators[1] as a more robust alternative to the benchmarking dataset generated from the GIAB consortium[2]. We outline the steps taken to obtain a somatic-only dataset by applying three rigorous filters to the truth dataset. Additionally, we provide details on the filters applied to the query Mutect2 dataset (VCF file containing variants identified by the caller) for comparison purposes. Finally, we present the results of the benchmarking analysis, including precision rates and false discovery rates (FDR).

Filtering the truth dataset to obtain a somatic-only dataset

The validated "Platinum" truth dataset, containing all curated germline and somatic calls derived from sample NA12878, along with the corresponding confident regions interval file, was obtained from the original publication[1].
Following established protocols[3] germline variants from the truth dataset were filtered out by querying public variation databases for common variants (>1% minimum allele fraction, MAF). The same databases used in our breast cohort were employed: 1000 Genomes Phase 3 data, GnomAD, and the Alfa Frequency Aggregator (see main method section). A reduction of approximately 99% in the number of calls was observed after applying these filters, with a better performance than what previously cited using older versions of the databases[3], as expected.
Secondly, analogous to our approach in our breast cohort, variants in regions known to be difficult to map, such as telomeric and centromeric regions, as well as repeated elements identified by RepeatMasker were removed (see main method section). Although these filtering steps may not significantly affect the rate of somatic mutations, they enhance confidence in the quality of the variants and remove erroneous calls due to alignment challenges[4].
Next, a "panel of normals" (PON) was created. While it is recognized in the literature that a PON consisting of 200-400 individuals is necessary for removing germline variants and achieving the accuracy of a matched normal sample[5], a PON was generated using unrelated samples from our breast study. Combined with the aforementioned filters, this PON can still provide valuable insights into removing sequencing artifacts specific to BGI platforms. For this benchmarking analysis, a PON consisting of all 58 breast samples that we previously sequenced was generated; these samples are all unrelated to the GIAB sample NA12878 but similarly sequenced on a BGI platform. Any variants present in the PON were removed from the truth dataset to ensure the removal of sequencing artifacts.

Filtering the query Mutect2 dataset

To evaluate the quality of the filters applied to our breast cohort in identifying calls, the same strategy described in the main method section was applied to the query (Mutect2) dataset for benchmarking purposes. The query dataset was generated by running Mutect2 with standard settings using the only BGI-sequenced bam file of NA12878 available from the GIAB consortium (available at https://giab.s3.amazonaws.com/data/NA12878/BGISEQ500/standard_library/), which was previously aligned to hg19 and submitted by BGI[6] as "tumour sample", and one breast sample from our study (E65) as "normal sample". The output calls were then filtered using FilterMutectCalls.
Similar to the truth dataset, the three filters to remove germline variants from the query dataset were applied. Thus, the query dataset was subjected to the filters based on: a) Frequency

databases, b) Low mappability regions. The further restriction of the files to the confident regions of the genome (derived from the supplied bed file) do not have any noticeable effect after the removal of repetitive and low mappability regions, as expected. c) PON.
Furthermore, a fourth filter based on read depth, which was also employed in our breast cohort, was used:

- Minimum number of reads for the "tumor" (NA12878 / GIAB sample) = 10,
- Minimum number of reads for the "tumor" alternate allele = 5,
- Minimum number of reads for the "normal" (E65 / breast sample) = 10,
- Required number of reads for the "normal" alternate allele = 0.

A reduction of approximately 99% in the number of calls in the truth dataset was obtained through our filtration process (**Supplementary Fig. 4a**).

Comparison of the somatic truth and query datasets

To compare the filtered somatic truth and query datasets, all variants were intersected using the R package dplyr (v1.1.2) and, in parallel, the script som.py from the Illumina hap.py (v0.3.9) package, as previously recommended[7].
The resulting values obtained from this comparison are shown in **Supplementary Fig. 4b**: 17,346 true positives (TP) comprising 1,223 indels and 16,123 SNVs; 1,152 false positives (FP) comprising 69 indels and 1,032 SNVs.
These values indicate a precision rate of 0.94 and a false discovery rate (FDR) of 0.06.

SUPPLEMENTARY REFERENCES

1 Eberle, M. A. *et al.* A reference data set of 5.4 million phased human variants validated by genetic inheritance from sequencing a three-generation 17-member pedigree. *Genome Res* **27**, 157-164 (2017). https://doi.org:10.1101/gr.210500.116

2 Zook, J. M. *et al.* Integrating human sequence data sets provides a resource of benchmark SNP and indel genotype calls. *Nat Biotechnol* **32**, 246-251 (2014). https://doi.org:10.1038/nbt.2835

3 Dou, Y., Gold, H. D., Luquette, L. J. & Park, P. J. Detecting Somatic Mutations in Normal Cells. *Trends Genet* **34**, 545-557 (2018). https://doi.org:10.1016/j.tig.2018.04.003

4 Tarailo-Graovac, M. & Chen, N. Using RepeatMasker to identify repetitive elements in genomic sequences. *Curr Protoc Bioinformatics* **Chapter 4**, 4 10 11-14 10 14 (2009). https://doi.org:10.1002/0471250953.bi0410s25

5 Hiltemann, S., Jenster, G., Trapman, J., van der Spek, P. & Stubbs, A. Discriminating somatic and germline mutations in tumor DNA samples without matching normals. *Genome Res* **25**, 1382-1390 (2015). https://doi.org:10.1101/gr.183053.114

6 Huang, J. *et al.* A reference human genome dataset of the BGISEQ-500 sequencer. *Gigascience* **6**, 1-9 (2017). https://doi.org:10.1093/gigascience/gix024

7 Krusche, P. *et al.* Best practices for benchmarking germline small-variant calls in human genomes. *Nat Biotechnol* **37**, 555-560 (2019). https://doi.org:10.1038/s41587-019-0054-x
