# Supplementary material for: The mutational landscape of the adult healthy parous and nulliparous human breast
Source: Nat Commun. 2023 Sep 6;14:5136. doi: 10.1038/s41467-023-40608-z (PMC10482899; doi:10.1038/s41467-023-40608-z)
Supplement: Supplementary file 2 — Description of Additional Supplementary Files [file 41467_2023_40608_MOESM2_ESM.pdf]

Description of Additional Supplementary Files

File Name: Supplementary Data 1
Description: **Clinical information of the samples used in the study**
For each patient, the table reports the corresponding identification label of the epithelial (Ep) and stromal (St) samples. The table also shows relevant clinical information, such as the age of the individual at the time of tissue collection (Age); the parity status; the number of gravidities (G) and number of full-term pregnancies (P); the age of the individual at the time of parturition of the first child (Age 1st birth); the number of years between first childbirth and tissue collection (Gap 1st birth donation); body mass index (BMI); whether the patient has a family history of breast cancer (BC) or ovarian cancer (OC).

File Name: Supplementary Data 2
Description: **Quality control of sequencing**
The table presents sequencing metrics for each sample. The metrics include the percentage of clean reads with a quality score of at least Q20, the percentage of clean reads with a quality score of at least Q30, the GC content percentage, the mapping rate to the reference genome, the average sequencing depth, and the percentage of bases covered at least 20X depth.

File Name: Supplementary Data 3
Description:**. Mutation burden in the healthy breast**
Summary of the number of nucleotide substitutions and insertion/deletions (INS/DEL) for each epithelial (top table) and stromal (bottom table) sample. The total and the median for each dataset is summarised at the end of each table. Additional relevant clinical information is reported: age of the individual at the time of tissue collection (Age); the parity status; the age of the individual at the time of parturition of the first child (Age 1st birth).

File Name: Supplementary Data 4
Description: **Copy number variations**
Summary of the copy number (CN) variations for the identified sample. Each sample identification is reported as sample number and the analysed component (Ep = Epithelium, St = Stroma). The table includes information such as the CytoBand location, the chromosome, the start and end positions of the variation, the total CN value and the associated genes.

File Name: Supplementary Data 5
Description: **Mutations in breast cancer-associated genes**
The tables present mutations in breast cancer-associated genes, in the epithelial and in the stromal samples. The subset of missense mutations is also shown in the bottom two tables. Each row provides detailed information for each mutation, with the following columns: Hugo Symbol, official gene symbol provided by the Human Genome Organization (HUGO) for the gene affected by the mutation; Chromosome, the chromosome where the mutation is located; Start and End Position, the genomic coordinate of the mutation's start and end position, respectively; Variant Classification, the type of mutation or variant; Variant Type, the specific type of variant; Reference Allele, he plus strand reference allele at this position. Includes the deleted sequence for a deletion or "-" for an insertion; Alt Seq Allele, the observed allele in the tumor sample at the mutation site; dbSNP RS, the rs-IDs from the database, "novel" if not found;

Sample, the name of the tumor sample; Donor, the patient's name, HGVSp Short, the protein sequence of the variant in Human Genome Variation Society recommended format, using 1-letter amino-acid codes. "p.=" signifies no change in the protein; Transcript ID, Ensembl ID of the transcript affected by the variant; VAF, Variant Allele Frequency; Consequence, the functional consequence of the mutation; BIOTYPE, the type of biotype affected by the mutation; IMPACT, the impact modifier for the consequence type, defined by the VEP software; SIFT, The Sorting Intolerant from Tolerant algorithm score for the impact of the mutation on protein function; PolyPhen, Polymorphism Phenotyping algorithm score for the impact of the mutation on protein structure and function; Compartment, the cellular compartment where the mutation was identified; Age resection, age of the individual at the time of tissue collection; Parity, parity status of the individual; Age birth, age of the individual at the time of the first childbirth (NA, if not applicable).
