# Supplementary material for: The mutational landscape of the adult healthy parous and nulliparous human breast
Source: Nat Commun. 2023 Sep 6;14:5136. doi: 10.1038/s41467-023-40608-z (PMC10482899; doi:10.1038/s41467-023-40608-z)
Supplement: Supplementary file 4 — Reporting Summary [file 41467_2023_40608_MOESM4_ESM.pdf]

nature portfolio

Corresponding author(s): Biancastella Cereser; Justin Stebbing

Last updated by author(s): Jul 24, 2023

Reporting Summary

Nature Portfolio wishes to improve the reproducibility of the work that we publish. This form provides structure for consistency and transparency in reporting. For further information on Nature Portfolio policies, see our Editorial Policies and the Editorial Policy Checklist.

Statistics

For all statistical analyses, confirm that the following items are present in the figure legend, table legend, main text, or Methods section.

n/a	Confirmed	
☐	☒	The exact sample size (*n*) for each experimental group/condition, given as a discrete number and unit of measurement
☐	☒	A statement on whether measurements were taken from distinct samples or whether the same sample was measured repeatedly
☐	☒	The statistical test(s) used AND whether they are one- or two-sided<br>*Only common tests should be described solely by name; describe more complex techniques in the Methods section.*
☐	☒	A description of all covariates tested
☒	☐	A description of any assumptions or corrections, such as tests of normality and adjustment for multiple comparisons
☐	☒	A full description of the statistical parameters including central tendency (e.g. means) or other basic estimates (e.g. regression coefficient) AND variation (e.g. standard deviation) or associated estimates of uncertainty (e.g. confidence intervals)
☐	☒	For null hypothesis testing, the test statistic (e.g. *F*, *t*, *r*) with confidence intervals, effect sizes, degrees of freedom and *P* value noted<br>*Give P values as exact values whenever suitable.*
☒	☐	For Bayesian analysis, information on the choice of priors and Markov chain Monte Carlo settings
☒	☐	For hierarchical and complex designs, identification of the appropriate level for tests and full reporting of outcomes
☐	☒	Estimates of effect sizes (e.g. Cohen's *d*, Pearson's *r*), indicating how they were calculated

Our web collection on statistics for biologists contains articles on many of the points above.

Software and code

Policy information about availability of computer code

Data collection	Image processing from sequencing data using the proprietary BGISEQ-500 platform software (Zebracall process version 0.5.0.13875) that is maintained, installed and distributed by Beijing Genomic Institute (Shenzhen, Guangdong, China)
Data analysis	Alignment of the reads and calling were carried out by the bioinformatics team at the Beijing Genomic Institute, following the guidelines of the Broad Institute Genome Analysis Toolkit (GATK, https://www.broadinstitute.org/gatk/guide/best-practices). Briefly, cleaned data for each sample were mapped to the human reference genome (GRCh37/HG19) with Burrows-Wheeler Aligner (v0.7.12), and duplicated reads were removed by Picard-tools (v1.118) with default settings.<br><br>Somatic short mutations, including SNV, small insertions, and deletions, were called via local assembly of haplotypes with Mutect2 (v4.1.4.1, https://gatk.broadinstitute.org/hc/en-us/sections/360007458971-4-1-4-1), the performance of which was previously established using synthetic whole-genome sequencing, with an adaptation of the "tumor with matched normal" function. To report both stroma- and epithelium-only mutations, the analysis therefore run as "epithelium vs stroma" and "stroma vs epithelium". Default settings for tumor-normal pairs were used. Called variants were successively filtered with FilterMutectCalls (v4.1.4.1, https://gatk.broadinstitute.org/hc/en-us/sections/360007458971-4-1-4-1), and only mutations categorized with PASS, representing confidently called somatic mutations, were used in the downstream analysis. VCF files were converted to Mutation Annotation Format (MAF) files according to National Cancer Institute specifications using Mskcc/vcf2maf (v1.6.17), adapting the format to include epithelial and stromal barcodes instead of tumor and normal barcodes, respectively. Annotated mutations were then further filtered for (a) the total number of reads > 10; the mutated number of reads > 5; variant allele frequency (VAF) > 0.02%; no reads in the control compartment; (b) removal of artifacts stemming from sex chromosome homology and repetitive regions (RepeatMasker 4.1.1, http://www.repeatmasker.org/); (c) frequency > 0.1% in the Genome Aggregation Database (gnomAD, V2.1.1, exome and genome samples, and gnomAD V3.1 https://gnomad.broadinstitute.org/), in the 1000 Genome database (https://www.internationalgenome.org/) and ALFA: Allele Frequency Aggregator (https://www.ncbi.nlm.nih.gov/snp/docs/gsr/alfa/

ALFA_20200227123210); (d) removal of possible germline mutations not detected with FilterMutectCalls present in a minimum of 2 samples from the pooled panel of matching compartment (epithelium or stroma).
MAF were visualized and summarized using the R Bioconductor package, maftools (v1.8.10) when necessary before proceeding to detailed analysis.

To ensure the minimal occurrence of false positives derived from either the presence of common polymorphisms or from the sequencing technology, thus increasing the accuracy of somatic variant calling in BGI-sequenced data using Mutect2, our calling pipeline was further benchmarked on a BGI-sequenced sample (NA12878) deposited in the Genome In A Bottle (GIAB) consortium database using the “platinum” truth variant catalogue previously generated filtered to obtain only somatic calls. The validated “Platinum” truth dataset, containing all curated germline and somatic calls derived from sample NA12878, along with the corresponding confident regions interval file, was obtained from the original publication (https://doi.org:10.1101/gr.210500.116). Germline variants from the truth dataset were filtered out by querying public variation databases for common variants (>1% minimum allele fraction, MAF). The same databases used in our breast cohort were employed: 1000 Genomes Phase 3 data, GnomAD, and the Alfa Frequency Aggregator (see main method section). Secondly, analogous to our approach in our breast cohort, variants in regions known to be difficult to map, such as telomeric and centromeric regions, as well as repeated elements identified by RepeatMasker were removed. Next, a "panel of normals" (PON) was created. A PON consisting of all 58 breast samples that we previously sequenced was generated; these samples are all unrelated to the GIAB sample NA12878 but similarly sequenced on a BGI platform. Any variants present in the PON were removed from the truth dataset to ensure the removal of sequencing artefacts. To evaluate the quality of the filters applied to our breast cohort in identifying calls, the same strategy described in the main method section was applied to the query (Mutect2) dataset for benchmarking purposes. The query dataset was generated by running Mutect2 with standard settings using the only BGI-sequenced bam file of NA12878 available from the GIAB consortium (available at https://giab.s3.amazonaws.com/data/NA12878/BGISEQ500/standard_library/), which was previously aligned to hg19 and submitted by BGI (https://doi.org:10.1093/gigascience/gix024) as “tumour sample”, and one breast sample from our study (E65) as “normal sample”. The output calls were then filtered using FilterMutectCalls.
Similar to the truth dataset, the three filters to remove germline variants from the query dataset were applied. Thus, the query dataset was subjected to the filters based on: a) Frequency databases, b) Low mappability regions. c) PON. Furthermore, a fourth filter based on read depth, which was also employed in our breast cohort, was used:
- Minimum number of reads for the "tumor" (NA12878 / GIAB sample) = 10,
- Minimum number of reads for the "tumor" alternate allele = 5,
- Minimum number of reads for the "normal" (E65 / breast sample) = 10,
- Required number of reads for the "normal" alternate allele = 0.

To compare the filtered somatic truth and query datasets, all variants were intersected using the R package dplyr (v1.1.2) and, in parallel, the script som.py from the Illumina hap.py (v0.3.9) package.

Obtained variants were categorized into synonymous and non-synonymous according to the impact on protein-coding of each variant (Ensembl IMPACT rating). Non-synonymous variants with high or moderate IMPACT rating were: Frameshift deletions, Frameshift insertions, Nonsense mutations, Nonstop mutations, Splice sites, Translation start sites, In-frame deletions, In-frame insertions, Missense mutations. Synonymous mutations with low or modifier IMPACT were: 3' Flank, 5' Flank, 3' UTR, 5' UTR, Intron, IGR, Splice regions, RNA, Silent mutations.
Downstream analysis was conducted in R and all figures were produced using the package ggplot2 (v3.3.0), unless otherwise specified. Linear regression analysis provided estimates for the change of the mutational burden with age.

The list of identified breast cancer (BC) drivers included in our analysis were taken from the paper “Landscape of somatic mutations in 560 BC whole-genome sequences” by Nik-Zainal et al., Nature, 2016. Individual driver events were taken from the same manuscript and found in Table S14: Driver events by mutation type 01052015.v2. Annotations on the oncogenic effects of the proteins were taken from OncoKB™ (version updated in October 2022)

The contribution of known mutational SBS signatures from the COSMIC database was determined using the R Bioconductor package MutationalPatterns (v3.0.1). Briefly, VCF files were imported as GRanges object and the sequence context was derived from the imported Reference Genome hg19, installed with the R Bioconductor package BSGenome (v1.58.0). To avoid overfitting of signatures, the function fit_to_signatures_strict was implemented with a final cutoff of 0.005.

Copy-number variants were called by cn.MOPS (v1.44.0) , using the getReadCountsFromBAM function with a window length parameter WL = 20000, according to the authors’ recommendations. The function referencecn.mops was applied to allow a modified “Tumor vs. Normal” setting, where the tumor was substituted with normal epithelium, and matched normal was substituted with matched normal stroma.
Variants were annotated with AnnotSV (v3.1) with the following settings to call and benign entries: benignAF = 0.01%, minTotalNumber = 500. Benign variants are automatically included by AnnotSV from the following datasets: ClinVar, ClinGen, Database of Genomic Variants (dgv, nsv or esv), gnomAD, Deciphering Developmental Disorders, 1000 Genomes, Ira M. Hall’s lab, Children’s Mercy Research Institute. The remaining parameters were set to default. AnnotSV also allowed the filtration of putative calls present in problematic regions of the genome (ENCODE blacklist). All putative regions were further manually inspected on Integrative Genomics Viewer (IGV, v2.4.19). The reads were compared to those of the matched samples, and true positive calls were validated on the presence of drops in the coverage compared to both matched control and neighboring regions, split-reads or paired-end abnormal signal.

All tools employed and associated versions are described above.

For manuscripts utilizing custom algorithms or software that are central to the research but not yet described in published literature, software must be made available to editors and reviewers. We strongly encourage code deposition in a community repository (e.g. GitHub). See the Nature Portfolio guidelines for submitting code & software for further information.

Data

Policy information about availability of data

All manuscripts must include a data availability statement. This statement should provide the following information, where applicable:

- Accession codes, unique identifiers, or web links for publicly available datasets
- A description of any restrictions on data availability
- For clinical datasets or third party data, please ensure that the statement adheres to our policy

All data is available in the main text or Supplementary materials. Aligned reads (BAM format) are available via the European Genome-Phenome Archive with accession number EGAS00001004672. The raw sequencing data are available under restricted access due to data privacy laws; access can be requested to the Data Access Committee as detailed here: https://ega-archive.org/access/data-access. The GRCh37/hg19 version of the human reference genome (http://genome.ucsc.edu) has been employed in this study.

Research involving human participants, their data, or biological material

Policy information about studies with human participants or human data. See also policy information about sex, gender (identity/presentation), and sexual orientation and race, ethnicity and racism.

Reporting on sex and gender	This study aimed to investigate the impact of parous and nulliparous status on mutation profiles, so female donors provided the pertinent samples to address this key aspect of research in the mammary gland. Because of this, our study exclusively included female patients and no further sex and gender-based analysis was performed. The decision to focus solely on female individuals was driven by the biological relevance of breast tissue in the context of reproductive history. The biological sex of the participant was obtained from the clinical records of the Komen Tissue Bank, which is the provider of the samples.
Reporting on race, ethnicity, or other socially relevant groupings	Race, ethnicity, or other socially relevant groupings are not annotated and have not been considered in this study.
Population characteristics	This study includes samples of normal breast from 29 women, aged 23 to 76.
Recruitment	The sample cohort was defined by cancer-free women who donated breast tissue biopsy specimens to the Susan G. Kamen Tissue Bank.<br>Our methods included the use of laser-capture microdissection of the epithelium, so we selected samples characterised by a high proportion of epithelial cells (determined by pathologists from the tissue bank after evaluation of H&E).<br>The population was selected for an approximately equal distribution of parous and nulliparous women of comparable age. Subject demographic, BMI, medication history and breast cancer risk factors were collected fusing a questionnaire administered by the Susan G. Kamen Tissue Bank.<br>The cellular architecture of the human mammary gland is affected by a variety of factors, the main one being hormonal regulation. To minimize inter-sample variability, we requested samples from individuals without previous use of hormonal contraceptive or hormonal replacement therapies.<br>We wanted to analyze the contribution of pregnancy in developing breast cancer, so we collected individuals without any previous or ongoing cancer, to eliminate any underlying factors which may affect the somatic mutational burden in the breast tissue. We cross-checked this by carefully checking the medication history of each recruited patient.<br>Breast cancer may have a hereditary component, associated with mutations in BRCAl and BRCA2. Our samples do not carry any pathogenic mutations in these genes.<br>Breast cancer risk has been associated with multi parity. While studies are still discordant on this, we decided to include mostly uniparous women in the parous group (15/17 women were uniparous).
Ethics oversight	Normal breast tissue samples, constituted by one biopsy per donor, from patients not affected by cancer were obtained from the Susan G. Komen Tissue Bank at IU Simon Cancer Center, following patient consent and approval from the local research ethics committee (IRB Protocol number is 1011003097). Human samples used in this research project were stored in the Imperial College Healthcare Tissue Bank (ICHTB) with sub-collection ONC_JS_18_001. ICHTB is supported by the National Institute for Health Research (NIHR) Biomedical Research Centre based at Imperial College Healthcare NHS Trust and Imperial College London. ICHTB is approved by Wales REC3 to release human material for research (REC 17/WA/0161). This work was performed following the ethical principles in the Declaration of Helsinki.

Note that full information on the approval of the study protocol must also be provided in the manuscript.

Field-specific reporting

Please select the one below that is the best fit for your research. If you are not sure, read the appropriate sections before making your selection.

- [x] Life sciences
- [ ] Behavioural & social sciences
- [ ] Ecological, evolutionary & environmental sciences

For a reference copy of the document with all sections, see nature.com/documents/nr-reporting-summary-flat.pdf

Life sciences study design

All studies must disclose on these points even when the disclosure is negative.

Sample size	In the absence of a formal prior sample size calculation, our study utilized a total of 58 samples from 29 donors, thoughtfully selected to ensure representation across age groups and reproductive history. The cohorts were as follows: 1) Less than or equal to 24 years old: 3 parous (P) and 3 nulliparous (NP); 2) Ages 25-34: 5 P and 3 NP; 3) Ages 35-49: 5 P and 4 NP; 4) Over 50 years old: 4 P and 2 NP. Despite the challenge in sourcing this unique cohort, the modest sample size allows for valuable observations, offering promising implications for our understanding of breast cancer etiology and warranting further exploration in future research.
Data exclusions	No samples were excluded. In the last extended figure of the paper, we included an additional subset of 4 parous samples which were excluded by the main study, because their age of tissue collection was >10 years from the age of pregnancy. The reasons for doing this are clearly described within the paper.
Replication	This work is based on human tissue, therefore exact technical replication is not possible. Each sample is an independent entity. We extracted both epithelial and stromal DNA from approximately 30 serial sections per sample. After microdissection, each cellular compartment and each sample was processed separately to avoid cross-contamination. Library preparation and sequencing was performed in batches.
Randomization	This study includes samples of parous and nulliparous female breast, without randomization.
Blinding	The study does not involve any scoring or analysis which could be affected by investigator's bias, therefore no blinding was necessary. All analysis was performed on all samples, and further categorized by parity groups.

Reporting for specific materials, systems and methods

We require information from authors about some types of materials, experimental systems and methods used in many studies. Here, indicate whether each material, system or method listed is relevant to your study. If you are not sure if a list item applies to your research, read the appropriate section before selecting a response.

Materials & experimental systems

n/a	Involved in the study
☒	☐ Antibodies
☒	☐ Eukaryotic cell lines
☒	☐ Palaeontology and archaeology
☒	☐ Animals and other organisms
☒	☐ Clinical data
☒	☐ Dual use research of concern
☒	☐ Plants

Methods

n/a	Involved in the study
☒	☐ ChIP-seq
☒	☐ Flow cytometry
☒	☐ MRI-based neuroimaging
